# Dual-phase ^18^F-florbetaben PET provides cerebral perfusion proxy along with beta-amyloid burden in Alzheimer’s disease

**DOI:** 10.1016/j.nicl.2021.102773

**Published:** 2021-07-24

**Authors:** Hai-Jeon Yoon, Bom Sahn Kim, Jee Hyang Jeong, Geon Ha Kim, Hee Kyung Park, Min Young Chun, Seunggyun Ha

**Affiliations:** a Department of Nuclear Medicine, Ewha Womans University, School of Medicine, Seoul, Republic of Korea; b Department of Neurology, Ewha Womans University School of Medicine, Republic of Korea; c Division of Psychiatry, Department of mental health care of older people, University College London, London, UK; d Division of Nuclear Medicine, Department of Radiology, Seoul St. Mary’s Hospital, College of Medicine, The Catholic University of Korea, Seoul, Republic of Korea

**Keywords:** ^18^F-florbetaben, Positron emission tomography, Dual-phase, R1, Alzheimer’s disease

## Abstract

**Background:**

This study investigated changes in brain perfusion and Aβ burden according to the progression of Alzheimer’s disease (AD) by using a dual-phase ^18^F-florbetaben (FBB) PET protocol.

**Methods:**

Sixty subjects, including 12 with Aβ-negative normal cognition (Aβ^−^NC), 32 with Aβ-positive mild cognitive impairment (Aβ^+^MCI), and 16 with Aβ-positive AD (Aβ^+^AD), were enrolled. A dynamic PET scan was obtained in the early phase (0–10 min, eFBB) and delayed phase (90–110 min, dFBB), which were then averaged into a single frame, respectively. In addition to the averaged eFBB, an R1 parametric map was calculated from the eFBB scan based on a simplified reference tissue model (SRTM). Between-group regional and voxel-wise analyses of the images were performed. The associations between cognitive profiles and PET-derived parameters were investigated.

**Results:**

Both the R1 and eFBB perfusion reductions in the cortical regions were not significantly different between the Aβ^−^NC and Aβ^+^MCI groups, while they were significantly reduced from the Aβ^+^MCI to Aβ^+^AD groups in regional and voxel-wise analyses. However, cortical Aβ depositions on dFBB were not significantly different between the Aβ^+^MCI and Aβ^+^AD groups. There were strong positive correlations between the R1 and eFBB images in regional and voxel-wise analyses. Both perfusion components showed significant correlations with general and specific cognitive profiles.

**Conclusion:**

The results of this study demonstrated the feasibility of dual-phase ^18^F-FBB PET to evaluate different trajectories of dual biomarkers for neurodegeneration and Aβ burden over the course of AD. In addition, both eFBB and SRTM-based R1 can provide robust indices of brain perfusion.

## 1 Introduction

Alzheimer’s disease (AD) is the most common type of dementia, accounting for an estimated 60% to 80% of dementia (As, 2018). AD is clinically characterized by progressive memory loss with functional impairments in frontal/executive, visuospatial, and language domains. The histopathological detection of extracellular β-amyloid (Aβ) plaques and intracellular neurofibrillary tangles (NFTs) in autopsied brain is still the gold standard for diagnosis of AD; however, postmortem studies have indicated that there is only a modest correspondence of autopsy results with the clinical diagnosis of AD (Khachaturian, 1985).

The research framework for AD diagnosis established by the National Institute on Aging and Alzheimer's Association (NIA-AA) proposed the so-called ATN classification system based on biomarker evidence of amyloid (A), tau (T), and neurodegeneration (N) (Jack et al., 2018). Of the ATN biomarkers, positron emission tomography (PET) can provide markers of amyloid deposition (A) and neurodegeneration (N). Amyloid deposition can be examined by using target specific radiotracers such as (Blomquist et al., 2008) C-Pittsburgh compound-B (Klunk et al., 2004); ^18^F-florbetapir (Johnson et al., 2013); ^18^F-flutemetamol (Nelissen et al., 2009), and ^18^F-florbetaben (Rowe et al., 2008). Neurodegeneration can be examined by demonstrating reduced metabolism in the temporoparietal cortex using ^18^F-fluorodeoxyglucose (^18^F-FDG) (Silverman, 2004). As a reflection of downstream neuronal injury, reduced perfusion in the temporoparietal cortex can be used as a proxy marker of neurodegeneration (Bradley et al., 2002).

Although the combination of amyloid PET and FDG PET scans can provide two main classes of ATN biomarkers, this approach is limited by its high cost and associated radiation exposure. Accumulating evidence has shown that the early time frames of dynamic amyloid PET are closely related to the first-pass influx rate (K1), which is strongly correlated with cerebral perfusion due to a high extraction fraction of lipophilic radiotracers in the brain (Forsberg et al., 2012, Blomquist et al., 2008, Chen et al., 2015, Tiepolt et al., 2016). Alternatively, the ratio of K1 to its reference region value (delivery rate, R1), which is an indicator of relative perfusion, can be obtained using the simplified reference tissue model (SRTM) method, whereas the kinetic modeling method to derive the K1 value requires concomitant arterial cannulation during dynamic PET scanning (Chen et al., 2015). The delivery rate R1 has shown a good correlation to ^18^F-FDG uptake, suggesting its potential use as a biomarker of neuronal dysfunction (Rodriguez-Vieitez et al., 2017, Hsiao et al., 2012, Joseph‐Mathurin et al., 2018). A recent study by Ottoy et al. demonstrated that the delivery rate R1 from kinetic modeling is robust over early-phase ^18^F-florbetapir PET for accurate representation of cerebral perfusion in AD (Ottoy et al., 2019).

Theoretically, perfusion can be derived from the earliest frames of dynamic PET scans, while amyloid deposition can be estimated from the later frames; thereby, complementary PET biomarkers can be obtained simultaneously using a dual-phase dynamic PET protocol. Such a dual-phase PET protocol would reduce patient cost and radiation exposure while increasing convenience. In this study, we aimed to investigate the feasibility of dual-phase ^18^F-florbetaben (FBB) PET for tracking both amyloid deposition and downstream neurodegeneration in three groups including subjects with Aβ-negative normal cognition (Aβ^−^NC), Aβ-positive mild cognitive impairment (Aβ^+^MCI), and Aβ-positive AD (Aβ^+^AD). For the early-phases of ^18^F-FBB PET (eFBB), the perfusion components of eFBB and STRM-based R1 were compared. We also examined the correlation between these imaging biomarkers and the severity of cognitive decline as measured by a standardized neuropsychological battery.

## 2 Materials and methods

### 2.1 Study population

Between November 2018 and December 2020, 60 subjects who completed dual-phase ^18^F-FBB PET scanning and T1-weighted magnetic resonance imaging (MRI) were retrospectively included in this study. Categorization into diagnostic groups was performed based on clinical history, neurological examinations, laboratory findings, neuropsychological test results, and neuroimaging studies including PET and MRI. Two expert PET readers (Y.H.J. and K.B.S.) visually assessed ^18^F-FBB PET data masked to all clinical information and rated Aβ-positivity (Barthel et al., 2011, Bullich et al., 2017). Briefly, the tracer uptake in four cortical regions (lateral temporal cortex, frontal cortex, parietal cortex, and posterior cingulate cortex/precuneus) was assessed according to the regional cortical tracer uptake (RCTU) system (1 = no uptake, 2 = moderate uptake, 3 = pronounced uptake). Then, the global uptake of the brain was assessed according to the brain amyloid plaque load (BAPL) system (1 = RCTU score 1 in each of the 4 brain regions, 2 = RCTU score 2 in any of the 4 brain regions and no RCTU score 3 in these regions, 3 = RCTU score 3 in at least one of the 4 brain regions). Finally, PET scans with BAPL scores of 2 and 3 were rated as Aβ-positive. The diagnostic criteria for MCI and AD were based on those proposed by the NIA-AA (Albert et al., 2011, McKhann et al., 2011). Finally, 12 Aβ-negative cognitively unimpaired subjects (Aβ^−^NC), 32 subjects with Aβ-positive MCI (Aβ^+^MCI), and 16 subjects with Aβ-positive AD (Aβ^+^AD) were classified. All subjects provided informed consent for PET imaging. This study was performed in accordance with the principles of the 1975 Declaration of Helsinki (2013 version) and approved by the Institutional Review Board of Ewha University Mokdong Hospital.

### 2.2 ^18^F-FBB PET imaging

^18^F-FBB (Neuraceq™) was manufactured by DuChemBio Co., Ltd. (Seoul, Korea) in accordance with the approval process of the Korean Ministry of Food and Drug Safety (MFDS) and delivered to our institutional PET center. All ^18^F-FBB PET/CT procedures were performed according to our institution’s established protocol. Dynamic PET images were acquired in three-dimensional (3D) list-mode using a dedicated PET/CT scanner (Biograph mCT, Siemens) after a bolus injection of 308.12 ± 10.93 MBq ^18^F-FBB over 10 min for the early phase (0–10 min post injection, eFBB) and over 20 min for the delayed phase (90–110 min post injection, dFBB). A spiral CT of the brain was acquired with CT parameters of 120 kV, 30 mAs, and a slice thickness of 1.0 mm. Data obtained from the CT scans were used to correct the attenuation for PET emission data. To minimize motion artifacts, the subject’s head was immobilized with a head holder and fixation equipment made of a vacuum cushion. Standard PET data obtained from dual-phase scans were reconstructed into a 128 × 128 matrix (voxel size: 3.18 × 3.18 × 2.02 mm3) using the built-in 3D ordered subset expectation maximization algorithm with 4 iterations, 12 subsets, and a 5-mm Gaussian filter and then averaged into single frames of eFBB and dFBB. In addition, 10-min list-mode data for the early-phase scan were reconstructed into 15 frames (6 × 5 s, 3 × 10 s, 4 × 60 s, 2 × 150 s) to calculate the SRTM-based R1 (Lammertsma and Hume, 1996).

### 2.3 Image analysis

For the quantitative analysis, dual-phase ^18^F-FBB (0–10 min, 90–110 min) and R1 PET-to-3D T1 MRI coregistration were performed initially for each subject separately using PMOD v4.0 (PMOD Technologies Ltd., Zurich, Switzerland). Voxel-wise parametric R1 maps were generated using PXMOD v4.0 with SRTM2 and a fixed subject-specific k2′ extracted from the regional kinetic modeling toolbox (PKIN) (Ottoy et al., 2019). SRTM2 was chosen over SRTM because the former model has been shown to reduce noise (Wu and Carson, 2002). Volumes of interest (VOIs) were delineated using an automated maximum probability atlas method (3 probability maps of gray matter, white matter, cerebrospinal fluid) for segmentation of each subject’s MRI and the Automated Anatomical Labeling (AAL) atlas in PMOD. Interframe motion correction was performed for early dynamic images. All VOIs included the cortical gray matter target regions (frontal, parietal, lateral temporal, anterior and posterior cingulate, and occipital cortices), and the reference region (cerebellum). Dividing the standardized uptake values (SUVs) of the different target regions by that of the reference region resulted in regional SUV ratios (SUVRs) for eFBB and dFBB. The composite value was defined as the arithmetic mean of the values of all target regions (Barthel et al., 2011).

### 2.4 Voxel-wise analysis

In addition to the VOI examination, a voxel-wise analysis of eFBB and R1 parametric images was performed using Statistical Parametric Mapping software (SPM12; Wellcome Department of Cognitive Neurology, London, UK) implemented in MATLAB R2017a (MathWorks Inc., Natick, MA). Each subject’s eFBB and R1 parametric image was coregistered to its T1 MRI. For spatial normalization, MR of each subject was segmented using the tissue probability map implemented in SPM12 after image-intensity nonuniformity correction, and then nonlinear transformation parameters were calculated between the tissues of native and Montreal Neurological Institute space. The transformation matrix was applied to each eFBB and R1 image, which had been coregistered to the T1 MRI. Finally, each eFBB and R1 image was smoothed using an 8-mm full-width at half-maximum Gaussian kernel. A voxel-wise two-sample *t*-test was used to compare the distribution patterns of both eFBB and R1 images through the continuum of AD, i.e. between Aβ^−^NC and Aβ^+^MCI and between Aβ^+^MCI and Aβ^+^AD. A voxel-wise Pearson correlation analysis was used to evaluate the correlations between eFBB and R1 images. The statistical significance was determined at *p* < 0.05 with a false discovery rate (FDR) correction and an extent cluster threshold of more than 100 voxels.

### 2.5 Neuropsychological assessments

Neuropsychological assessments were performed using a standardized neuropsychological battery called the Seoul Neuropsychological Screening Battery (SNSB) (Forsberg et al., 2012, Blomquist et al., 2008). The SNSB includes exams for attention, language, calculation ability, visuospatial skills, memory, frontal-executive function, and general cognition such as the Mini-Mental State Examination (MMSE) and Clinical Dementia Rating (CDR). Attention was evaluated with the digit span (forward and backward) tests; language was evaluated with the Korean version of the Boston Naming Test (K-BNT); calculation ability was evaluated with the total scores for addition, subtraction, multiplication, and division; visuospatial function was evaluated with the Rey-Osterrieth Complex Figure Test (RCFT); memory function was evaluated with immediate and delayed recall on the Seoul Verbal Learning Test (SVLT); and frontal/executive function was evaluated with the Controlled Oral Word Association Test (COWAT) test (animal, supermarket, and phonemic) and the Stroop word/color reading test. A standardized Z-score based on age-, sex-, and education-adjusted norms was used for the analysis.

### 2.6 Statistical analyses

All statistical analyses were performed using SPSS software version 26.0 (SPSS Inc., Chicago, IL, USA). Differences in demographics and cognitive profiles among the three diagnostic groups were assessed using Fisher’s exact test for categorical variables and the Kruskal-Wallis test for continuous variables because the variables were not normally distributed. Differences in SUVR and R1 values in each of the 6 target regions plus the composite region among the three groups were explored using the Kruskal-Wallis test. The statistical threshold of the post-hoc analyses were Bonferroni corrected: *p* < 0.05/7 considering 7 comparisons (the composite region and 6 target regions).

Pearson correlation analysis was used to evaluate the correlations between eFBB SUVR and R1 values. A partial correlation controlling for age, gender, years of education, and the CDR score was calculated to evaluate relationships between composite values and cognitive scores; the results were regarded as significant if *p* < 0.05. Differences between apolipoprotein E4 (APOE4) carriers and noncarriers were analyzed using the Mann-Whitney *U* test for each diagnostic group except Aβ^−^NC because all of the subjects were noncarriers.

## 3 Results

### 3.1 Participant characteristics

The general characteristics of the study participants are shown in Table 1. None of the subjects had a history of cardiovascular disease. The years of education in the Aβ^+^AD group was significantly lower than that in the Aβ^−^NC and Aβ^+^MCI groups. The MMSE, CDR and CDR-SB scores showed significant differences through the continuum of AD. The proportion of APOE4 positivity in the Aβ^+^MCI and Aβ^+^AD groups was significantly greater than that in the Aβ^−^NC group.

**Table 1:** General features and cognitive scores of subjects.

	Aβ^−^NC (n = 12)	Aβ^+^MCI (n = 32)	Aβ^+^AD (n = 16)	p value
Age, years	71 (66, 78)	73 (69, 79)	71 (63, 77)	0.353
Female, %	83.33%	59.40%	68.80%	0.102
DM, %	25.00%	15.60%	6.30%	0.384
HTN, %	50.00%	46.90%	56.30%	0.829
Smoking, %	8.00%	15.60%	12.60%	0.399
Alcohol, %	8.00%	25.00%	18.80%	0.249
Right-handed, %	100.00%	90.60%	93.30%	0.235
Education, years	12.0 (7.5, 14.0)	12.0 (9.8, 16.0)	7.5 (6.0, 9.8)	0.006*
APOE4 carrier†	0.00%	57.70%	70.00%	<0.001*
MMSE score	29.0 (27.0, 30.0)	26.0 (24.3, 28.0)	18.0 (11.3, 22.8)	<0.001*
CDR score	0.0 (0.0, 0.0)	0.5 (0.5, 0.5)	1.0 (0.6, 1.8)	<0.001*
CDR-SB score	0.0 (0.0, 0.0)	1.5 (0.5, 2.4)	6.0 (4.1, 9.8)	<0.001*

Values are reported as medians with interquartile ranges. P-values were calculated using the Kruskal-Wallis test. *P-values are significant at the 0.05 level. †APOE4 genotyping was available in 12 subjects in the Aβ^−^NC group, 26 subjects in the Aβ^+^MCI group, and 10 subjects in the Aβ^+^AD group. *Abbreviations:* Aβ^−^NC, Aβ-negative normal cognition; Aβ^+^MCI, Aβ-positive mild cognitive impairment; Aβ^+^AD, Aβ-positive Alzheimer’s disease with dementia; DM, diabetes mellitus; HTN, hypertension; APOE, apolipoprotein E; MMSE, Mini-Mental State Examination; CDR, Clinical Dementia Rating; CDR-SB, Clinical Dementia Rating Scale Sum of Boxes.

### 3.2 Visual comparison of eFBB and R1 images according to the continuum of AD

**Fig. 1:**
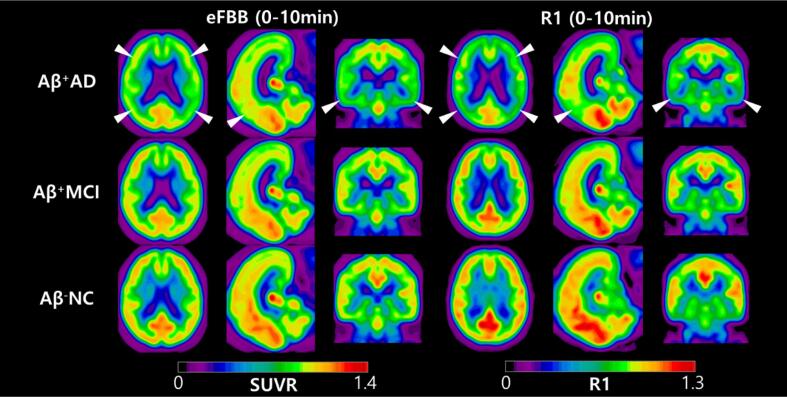
Group-averaged eFBB and R1 images. Axial, sagittal, and coronal views of average spatially normalized eFBB and R1 images of the Aβ^−^NC (n = 12), Aβ^+^MCI (n = 32), and Aβ^+^AD (n = 16) groups. White arrows indicate areas of perfusion reduction compared with the Aβ^−^NC and Aβ^+^MCI groups, including the frontal, parietal, temporal, and posterior cingulate cortices. *Abbreviations:* Aβ^−^NC, Aβ-negative normal cognition; Aβ^+^MCI, Aβ-positive mild cognitive impairment; Aβ^+^AD, Aβ-positive Alzheimer’s disease with dementia

Group-averaged eFBB images normalized to the cerebellum and R1 maps are shown in Fig. 1. Visually prominent perfusion reductions in the frontal, parietal, temporal, and posterior cingulate cortices were noted in Aβ^+^AD group compared with the Aβ^−^NC and Aβ^+^MCI groups. However, perfusion reductions were not prominent from Aβ^−^NC to Aβ^+^MCI. Both the eFBB and R1 maps demonstrated a similar pattern of perfusion reduction.

### 3.3 VOI-based and voxel-based analysis of eFBB images according to the continuum of AD

**Fig. 2:**
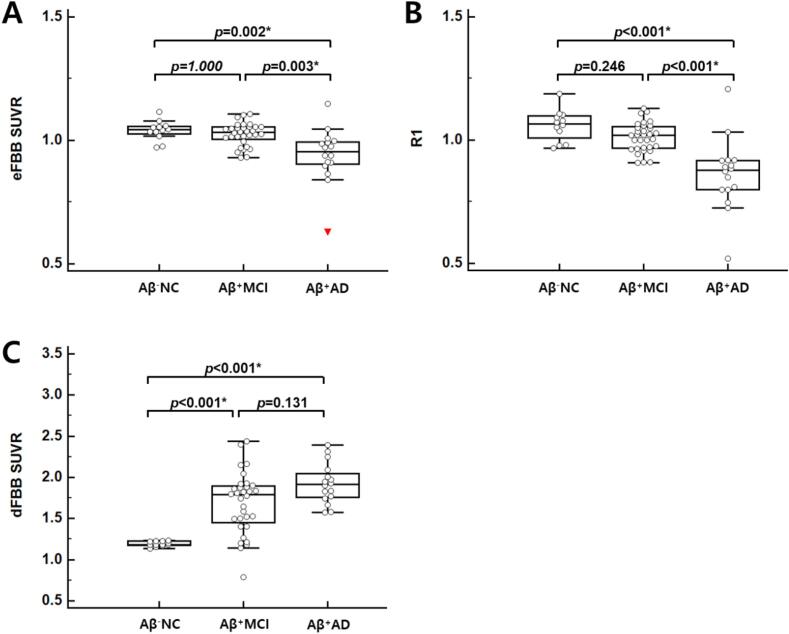
Composite eFBB SUVR, R1, and dFBB SUVR in the groups. Comparison of the eFBB SUVR, R1, and dFBB SUVR of the composite cortical regions according to three diagnostic groups. Both the eFBB (A) and R1 (B) images showed significant perfusion reductions in the Aβ^+^AD group compared with the Aβ^−^NC and Aβ^+^MCI groups. The composite dFBB SUVR (C) showed significant Aβ depositions in the Aβ^+^MCI and Aβ^+^AD groups compared with the Aβ^−^NC group (*; *p* < 0.05/7). *Abbreviations:* Aβ^−^NC, Aβ-negative normal cognition; Aβ^+^MCI, Aβ-positive mild cognitive impairment; Aβ^+^AD, Aβ-positive Alzheimer’s disease with dementia

The composite eFBB SUVR was significantly different according to the three diagnostic groups (*p* = 0.001, H = 14.963). A post-hoc pairwise comparison showed that the composite eFBB SUVR of the Aβ^+^AD group was significantly lower than that of the Aβ^−^NC and Aβ^+^MCI groups (*p* = 0.002 and *p* = 0.003; Fig. 2). However, there was no significant difference between the composite eFBB SUVR of the Aβ^−^NC and Aβ^+^MCI groups. For the target regions, the eFBB SUVR of the posterior cingulate, parietal, and lateral temporal cortices significantly differed in the three groups (*p* < 0.001, H = 18.639 for the posterior cingulate cortex; *p* = 0.001, H = 13.449 for the parietal cortex; *p* = 0.001, H = 14.797 for lateral temporal cortex). The regional eFBB SUVR of the other target cortices did not differ significantly in the three diagnostic groups. The eFBB SUVR data in the target cortices through the continuum of AD are summarized in Supplementary Fig. 1.

**Fig. 3:**
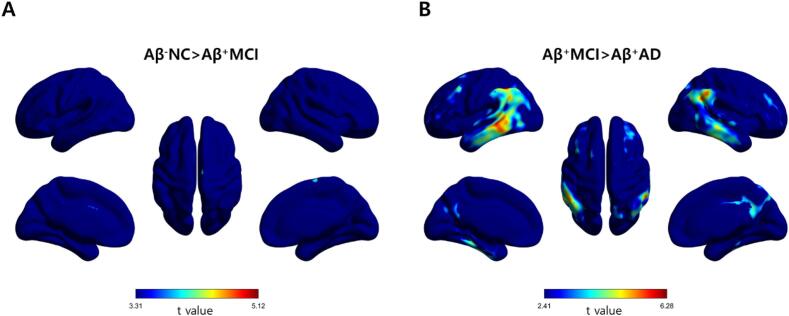
Statistical parametric maps from eFBB images. Statistical parametric maps of the hypoperfusion patterns obtained from eFBB (0–10 min) images in the Aβ^+^MCI group compared with the Aβ^−^NC group (A: uncorrected *p* < 0.001, *t* > 3.31) and in the Aβ^+^AD group compared with the Aβ^+^MCI group (B: FDR-corrected *p* < 0.05, *t* > 2.41). *Abbreviations:* Aβ^−^NC, Aβ-negative normal cognition; Aβ^+^MCI, Aβ-positive mild cognitive impairment; Aβ^+^AD, Aβ-positive Alzheimer’s disease with dementia

Fig. 3 shows the results of voxel-wise parametric mapping analysis for the different diagnostic groups. The eFBB perfusion reduction in the cortical regions was not significantly different between the Aβ^−^NC and Aβ^+^MCI groups (uncorrected *p* < 0.001, cluster extent of more than 100 voxels). The eFBB perfusion was significantly reduced in the frontal, temporal, and parietal cortical regions from the Aβ^+^MCI to the Aβ^+^AD groups (FDR-corrected *p* < 0.05, cluster extent of more than 100 voxels). The Aβ^+^AD group showed eFBB perfusion reduction in the frontal, temporal, and parietal cortical regions compared with the Aβ^−^NC group (FDR-corrected *p* < 0.05, cluster extent of more than 100 voxels; Supplementary Fig. 2).

### 3.4 VOI-based and voxel-based analysis of R1 parametric images according to the continuum of AD

The composite R1 was significantly different according to three diagnostic groups (*p* < 0.001, H = 24.311). A post-hoc pairwise comparison showed the composite R1 of the Aβ^+^AD group was significantly lower than that of the Aβ^−^NC and Aβ^+^MCI groups (*p* < 0.001; Fig. 2). However, there was no significant difference between the composite R1 of the Aβ^−^NC and Aβ^+^MCI groups. The R1 of all target regions except the anterior cingulate cortex significantly differed across the three groups (*p* < 0.001, H = 18.655 for frontal cortex; *p* < 0.001, H = 25.778 for posterior cingulate cortex; *p* < 0.001, H = 20.604 for parietal cortex; *p* < 0.001, H = 15.680 for occipital cortex; *p* < 0.001, H = 21.835 for lateral temporal cortex; *p* < 0.001). The R1 values in target cortices through the continuum of AD are summarized in Supplementary Fig. 3.

**Fig. 4:**
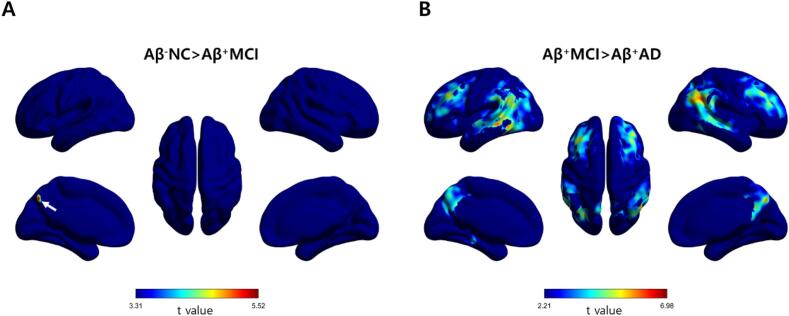
Statistical parametric maps from R1. Statistical parametric maps of the hypoperfusion patterns obtained from R1 (0–10 min) images in the Aβ^+^MCI group compared with the Aβ^−^NC group (A: uncorrected *p* < 0.001, *t* > 3.31) and in the Aβ^+^AD group compared with the Aβ^+^MCI group (B: FDR-corrected *p* < 0.05, *t* > 2.21). The arrow on the map of Aβ^−^NC > Aβ^+^ MCI indicates the area of perfusion reduction in the precuneus of the parietal cortex. *Abbreviations:* Aβ^−^NC, Aβ-negative normal cognition; Aβ^+^MCI, Aβ-positive mild cognitive impairment; Aβ^+^AD, Aβ-positive Alzheimer’s disease with dementia

Fig. 4 shows the results of voxel-wise analysis for the different diagnostic groups. The Aβ^+^MCI group showed perfusion reduction in the parietal cortex compared with the Aβ^−^NC group (uncorrected *p* < 0.001, cluster extent of more than 100 voxels). However, no voxels survived in the voxel-wise comparison between the Aβ^−^NC and Aβ^+^MCI group after correcting for the FDR. The R1 perfusion was significantly reduced in the frontal, temporal, and parietal cortical regions in subjects with Aβ^+^AD compared with those with Aβ^−^MCI (FDR-corrected *p* < 0.05, cluster extent of more than 100 voxels). The Aβ^+^AD group showed R1 perfusion reduction in the frontal, temporal, and parietal cortical regions compared with the Aβ^−^NC group (FDR-corrected *p* < 0.05, cluster extent of more than 100 voxels; Supplementary Fig. 4).

### 3.5 VOI-based analysis of dFBB images according to the continuum of AD

The composite dFBB SUVRs significantly different across the study groups (*p* < 0.001, H = 25.240). A post-hoc pairwise comparison showed the composite SUVR of the Aβ^+^MCI and Aβ^+^AD groups were significantly higher than those of the Aβ^−^NC group (*p* < 0.001; Fig. 2). However, there was no significant difference between the SUVR of the Aβ^+^MCI and Aβ^+^AD groups. The dFBB SUVRs of all target regions significantly differed across the three groups (*p* < 0.001, H = 27.916 for frontal cortex; *p* < 0.001, H = 24.975 for anterior cingulate cortex; *p* < 0.001, H = 19.379 for posterior cingulate cortex; *p* < 0.001, H = 25.343 for parietal cortex; *p* < 0.001, H = 22.107 for occipital cortex; *p* < 0.001, H = 24.808 for lateral temporal cortex; *p* < 0.001). The dFBB SUVRs in target cortices throughout the continuum of AD are summarized in Supplementary Fig. 5.

### 3.6 VOI-based and voxel-based correlations between R1 parametric images and eFBB images

**Fig. 5:**
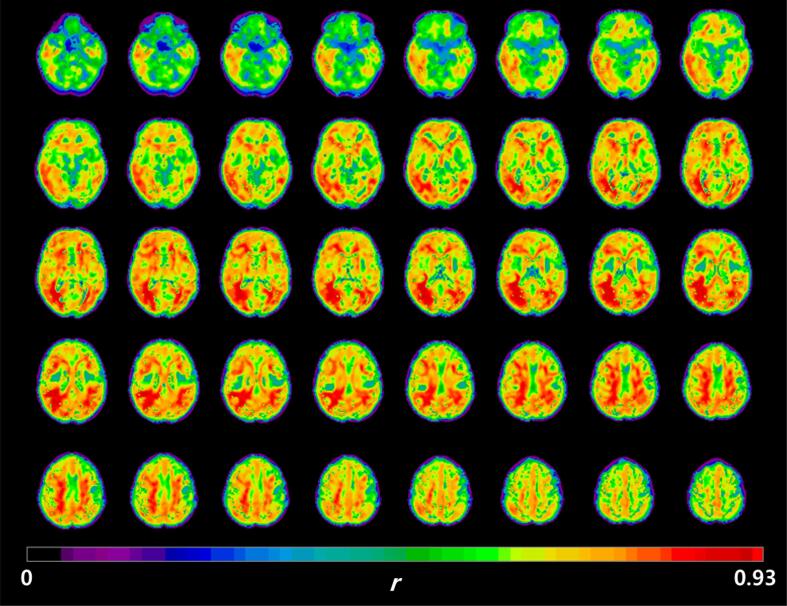
Correlation coefficient parametric maps between eFBB and R1 images. Correlation coefficient parametric maps showing a positive correlation between cerebral perfusion proxies after controlling for the CDR score (FDR-corrected *p* < 0.05, *r* > 0.27).

There were strong positive correlations between R1 and eFBB images (Pearson r ranging from 0.82 [frontal cortex] to 0.95 [lateral temporal cortex]; all *p* < 0.001). After controlling for the CDR score, these correlations were slightly weaker (Pearson r ranging from 0.75 [frontal cortex] to 0.91 [lateral temporal cortex]; all *p* < 0.001). The VOI-based correlation results are shown in Table 2. Voxel-wise correlations between cerebral perfusion proxies showed significant positive correlations after controlling for the CDR score (FDR-corrected *p* < 0.05, *r* > 0.27; Fig. 5).

**Table 2:** VOI-based correlation (Pearson r) between eFBB and R1 images.

	Before controlling for the CDR		After controlling for the CDR	
	r coefficient	p value	r coefficient	p value
Frontal cortex	0.821	<0.001*	0.754	<0.001*
Parietal cortex	0.910	<0.001*	0.881	<0.001*
Occipital cortex	0.844	<0.001*	0.842	<0.001*
Lateral temporal cortex	0.951	<0.001*	0.914	<0.001*
Anterior cingulate cortex	0.850	<0.001*	0.830	<0.001*
Posterior cingulate cortex	0.876	<0.001*	0.767	<0.001*
Composite	0.907	<0.001*	0.849	<0.001*

### 3.7 Relationship between neuropsychological tests and parameters derived from eFBB, R1, and dFBB images

**Fig. 6:**
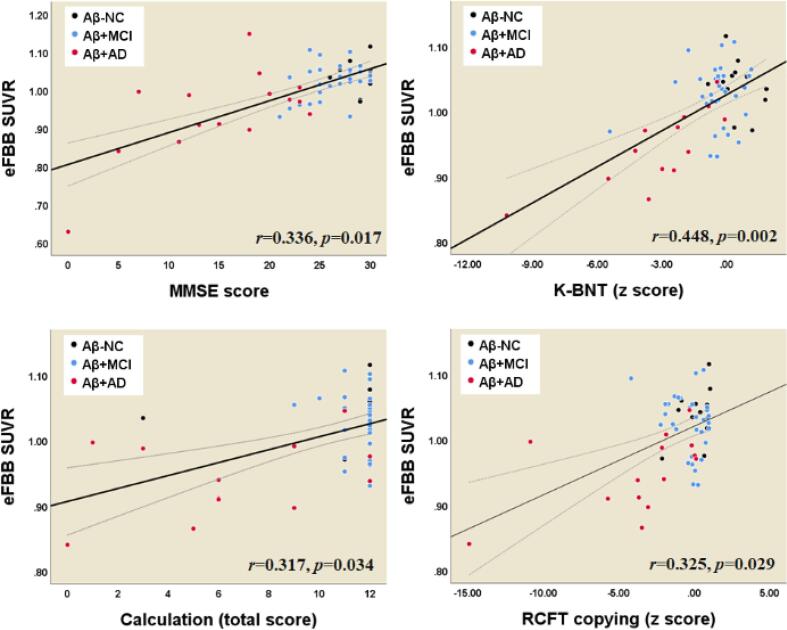
Correlations between composite SUVR values from eFBB and cognitive profiles. *Abbreviations:* Aβ^−^NC, Aβ-negative normal cognition; Aβ^+^MCI, Aβ-positive mild cognitive impairment; Aβ^+^AD, Aβ-positive Alzheimer’s disease with dementia; MMSE, Mini-Mental State Examination; K-BNT, Korean version of the Boston Naming Test; RCFT, Rey-Osterrieth Complex Figure Test.

**Fig. 7:**
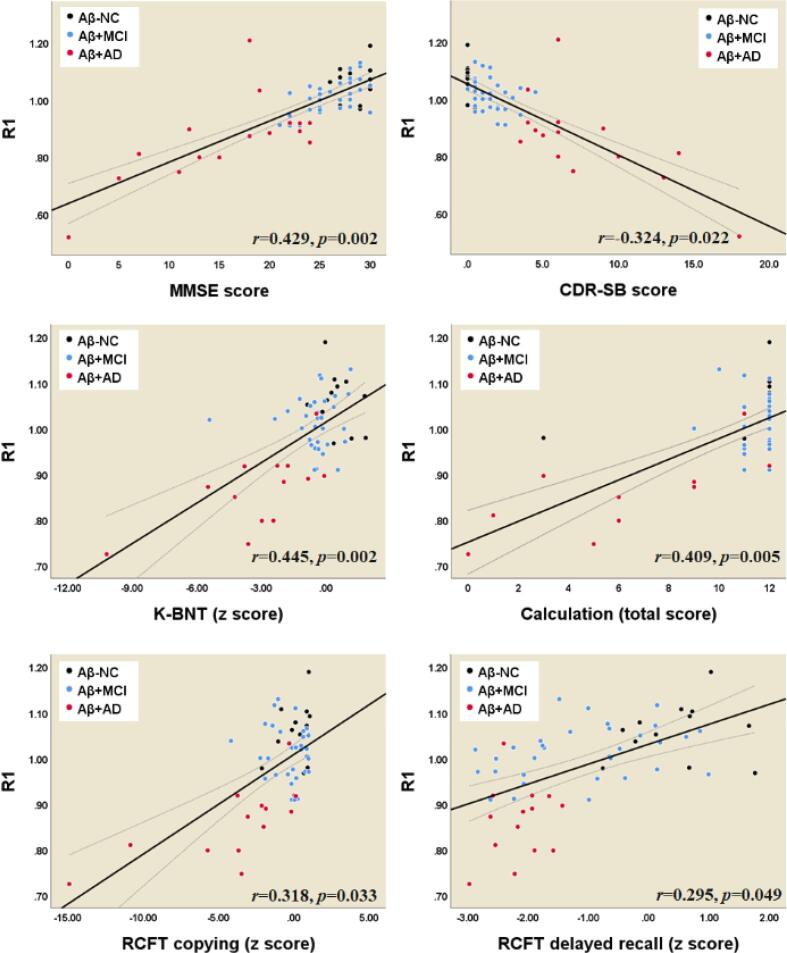
Correlations between composite R1 values and cognitive profiles. *Abbreviations:* Aβ^−^NC, Aβ-negative normal cognition; Aβ^+^MCI, Aβ-positive mild cognitive impairment; Aβ^+^AD, Aβ-positive Alzheimer’s disease with dementia; MMSE, Mini-Mental State Examination; CDR-SB, Clinical Dementia Rating Scale Sum of Boxes; K-BNT, Korean version of the Boston Naming Test; RCFT, Rey-Osterrieth Complex Figure Test.

**Fig. 8:**
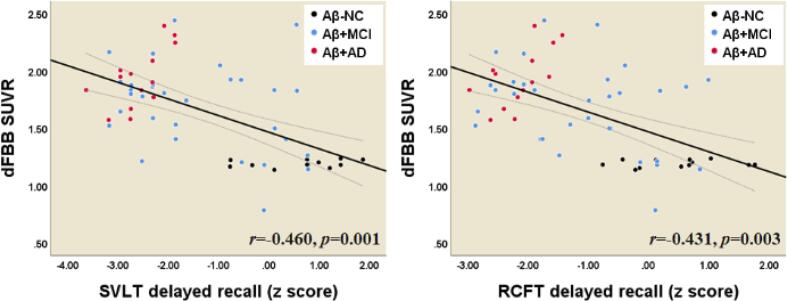
Correlations between composite SUVR values from dFBB and cognitive profiles. *Abbreviations:* Aβ^−^NC, Aβ-negative normal cognition; Aβ^+^MCI, Aβ-positive mild cognitive impairment; Aβ^+^AD, Aβ-positive Alzheimer’s disease with dementia; SVLT, Seoul Verbal Learning Test; RCFT, Rey-Osterrieth Complex Figure Test.

The perfusion derived from eFBB showed a significant positive correlation with the MMSE score (Fig. 6). In particularly, the perfusion derived from eFBB showed significant positive correlations with the z scores from the K-BNT and RCFT copy tests and with the calculation total score. The perfusion derived from R1 showed a significant positive correlation with the MMSE score and a negative correlation with the CDR-SB score (Fig. 7). In addition, R1 showed significant positive correlations with the z scores from the K-BNT test, the RCFT copy and delayed recall tests, and the calculation total score. The amyloid deposition as determined by dFBB showed significant negative correlations with the z scores from the RCFT and SVLT delayed recall tests (Fig. 8).

### 3.8 Comparison between APOE4 genotype subgroups

APOE4 genotyping was available in 12 subjects in the Aβ^−^NC group (all noncarriers), 26 subjects in the Aβ^+^MCI group (11 noncarriers and 15 carriers), and 10 subjects in the Aβ^+^AD group (3 noncarriers and 7 carriers). In the Aβ^+^MCI group, the MMSE score of APOE4 carriers was significantly lower than that of noncarriers (*p* = 0.002), while the CDR-SB score of APOE4 carriers was significantly higher than that of noncarriers (*p* = 0.008). The composite and all target regional eFBB SUVRs did not differ significantly according to APOE4 genotype. However, the composite R1 of APOE4 carriers was significantly lower than that of noncarriers (*p* = 0.004; Supplementary Fig. 6). Of target regions, the R1 of the posterior cingulate cortex was significantly lower in carriers than in noncarriers (*p* = 0.006; Supplementary Fig. 6). The composite dFBB SUVR of APOE4 carriers was significantly higher than that of noncarriers (*p* = 0.006, Supplementary Fig. 7). Of target regions, carriers showed significantly higher dFBB SUVRs than noncarriers in the frontal and anterior cingulate cortices (*p* = 0.002 for the frontal cortex, *p* = 0.001 for the anterior cingulate cortex; Supplementary Fig. 7). In the Aβ^+^AD group, there were no significant differences in variables between carriers and noncarriers.

## 4 Discussion

In this study, we evaluated the feasibility of dual-phase FBB PET for tracking both cerebral perfusion and amyloid deposition through the continuum of AD including Aβ^−^NC, Aβ^+^MCI, and Aβ^+^AD. The perfusion components derived from eFBB and R1 maps significantly differed across the three groups. The post-hoc comparison showed no significant difference between the Aβ^−^NC and Aβ^+^MCI groups, while there was significantly lower perfusion in the Aβ^+^AD group than in Aβ^−^NC and Aβ^+^MCI groups on both of early-phase maps. On the other hand, the Aβ deposition component derived from the delayed-phase of FBB PET showed significantly greater deposition in the Aβ^+^MCI and Aβ^+^AD groups than in the Aβ^−^NC group, while there were no significant differences between the Aβ^+^MCI and Aβ^+^AD groups. This alteration in the two components derived from dual-phase FBB PET showed a distinct pattern of initiation of Aβ deposition rather than perfusion reduction in the earlier stages of AD, which corresponds to the currently accepted hypothetical model of dynamic biomarkers of AD (Jack et al., 2010). Our cross-sectional design cannot fully demonstrate this, and longitudinal studies are needed to confirm dynamic changes of dual biomarkers.

Decreased cerebral perfusion during the course of neurodegeneration has been well documented in patients with AD (Thomas et al., 2019, Hirao et al., 2005). However, the results for MCI are still controversial since cerebral blood flow (CBF) measures show both hypoperfusion and hyperperfusion in brain subregions. This paradoxical hyperperfusion has been reported in the early and preclinical phases of AD and has been explained as a compensatory response to Aβ pathology (Fazlollahi et al., 2020). Johnson et al. reported that hypoperfusion of the parietal cortex in an MCI group compared with a healthy control group (Johnson et al., 2005). Dai et al. reported that a hypoperfusion of the posterior cingulate cortex and precuneus in an MCI group compared with a healthy control group; however, there was hyperperfusion of subcortical structures (Dai et al., 2009). Lin et al. evaluated cerebral perfusion according to the progression of AD by using the early phase of ^18^F-florbetapir PET obtained 1–6 min after injection (Lin et al., 2016). They further divided patients with MCI into three subgroups according to the degree of cognitive decline and reported that perfusion deficits started during late MCI; however, no significant perfusion deficits were found during early MCI. In this study, we also found that both perfusion components derived from eFBB and R1 maps were significantly reduced in typical cortical regions in subjects with Aβ^+^AD compared with those with Aβ^+^MCI, while there was no significant difference between the Aβ^−^NC and Aβ^+^MCI groups (Fig. 3, Fig. 4). We did not subdivide the MCI group, but the results showed that reduced cerebral perfusion occurred with definitive cognitive decline, as shown in the previous study. The lack of differences in cerebral perfusion between the Aβ^−^NC and Aβ^+^MCI groups seems to be due to the high proportion of subjects with early MCI in the Aβ^+^MCI group. In fact, when the criteria used by Lin et al. were applied to our Aβ^+^MCI group, there were no subjects corresponded to late MCI.

According to the hypothetical pathological cascade in AD, neurodegenerative biomarkers retain a closer correlation with clinical symptom severity than Aβ deposition (Jack et al., 2010). In this study, we found that decreased cerebral perfusion as evaluated by both eFBB and R1 maps were significantly correlated with deterioration of general cognition as evaluated by the MMSE and CDR-SB tests. In addition, the perfusion components were well correlated with the scores of specific neuropsychological tests for language, visuospatial function, and memory (Fig. 6, Fig. 7). We also observed that increased Aβ deposition as evaluated by dFBB was significantly correlated with cognitive decline. However, the perfusion component, which is a proxy for neurodegenerative biomarkers, showed a better relationship with cognitive performance than Aβ deposition. Of perfusion components, the R1-derived parameter showed significant correlations with a greater number of cognitive profiles than the eFBB-derived one.

Both eFBB and R1 maps are of clinical interest because PET tracers for Aβ provide information on cerebral perfusion in addition to Aβ deposition. Providing additional information about cerebral perfusion, which is a proxy indicator of neurodegeneration, may promote diagnostic accuracy while avoiding unnecessary radiation exposure and medical costs associated with a separate neuroimaging study (e.g., FDG PET or perfusion SPECT). The use of eFBB is preferred over R1 because labor-intensive kinetic modeling is required to obtain parametric maps. However, obtaining R1 is less labor-intensive than obtaining other kinetic parameters because it can be obtained using the SRTM method, which does not require invasive arterial sampling. Moreover, we found that R1 was correlated to a greater number of cognitive profiles than eFBB. On the voxel-wise analysis, R1 showed mild decreased perfusion in the precuneus of the parietal cortex from the Aβ^−^NC to Aβ^+^MCI groups, whereas eFBB failed to show any difference between the Aβ^−^NC and Aβ^+^MCI groups. However, the uncorrected analysis result should be interpreted cautiously, even when a low threshold is chosen. A recent study by Ottoy et al. evaluated perfusion components of early-phase ^18^F-florbetapir PET and SRTM-based R1 in comparison with (Hsiao et al., 2012)O-H_2_O PET, a gold standard for CBF, and also reported the robust value of R1 (Ottoy et al., 2019).

In a subgroup analysis comparing APOE4 carriers and noncarriers, we found patterns of lower cerebral perfusion and higher Aβ deposition in carriers than noncarriers in the Aβ^+^MCI group. A strong effect of APOE4 on Aβ deposition is consistent with the findings of previous studies (Gonneaud et al., 2016). For neurodegeneration, a deleterious effect of APOE4 on gray matter volume and glucose metabolism, especially in AD-sensitive cortical regions, has been reported (Paranjpe et al., 2019). Our findings support previous studies with FDG PET by presenting similar effects of APOE4 genotype on cerebral perfusion as a proxy marker of neurodegeneration. In the Aβ^+^AD group, there were no significant differences in perfusion proxies and Aβ deposition between carriers and noncarriers possibly because perfusion reduction and Aβ deposition had reached a plateau point.

There are several limitations to our study. First, we did not obtain full dynamic scans for 110 min. Studies have indicated that obtaining the early time frame is sufficient to evaluate perfusion due to the high extraction fraction of lipophilic radiotracers into the brain (Forsberg et al., 2012, Ottoy et al., 2019, Lin et al., 2016). Although a previous study by Heeman et al. suggested a dual-time window protocol of 0–30 and 90–110 min as an optimal one for accurate estimation of binding potential (BP_ND_, Aβ load), they observed only a small error in SRTM-derived R1 for 0–10 and 90–110 min, which would be negligible for practical applications. In this study, we estimated the SUVR for the delayed-phase scan rather than BP_ND_; thus, a 10-min acquisition for the early-phase scan would be sufficient to generate a reliable R1 estimation (Heeman et al., 2019). Second, we did not perform ^18^F-FDG PET or ^15^O-H_2_O PET as current standards for neurodegeneration or CBF measurement. Although direct comparison was not available in this study, previous studies have demonstrated that the value of early-phase amyloid PET as a proxy indicator of neurodegeneration or cerebral perfusion deficits (Ottoy et al., 2019). Third, we investigated the feasibility of dual-phase ^18^F -FBB PET for tracking both amyloid deposition and downstream neurodegeneration according to the continuum of AD, but we did not include the full disease spectrum because subjects with preclinical AD were not included in this study. Finally, the education level in the Aβ^+^AD group was significantly lower than that in the Aβ^−^NC and Aβ^+^MCI groups. A low education level is known to be related strongly to the risk of AD (Caamaño-Isorna et al., 2006). Education is considered to improve the cognitive reserve and neuropsychological task performance (Vadikolias et al., 2012, Matyas et al., 2019); thus, the low education level in the Aβ+AD group may be a confounding factor for the degree of correlation between PET biomarkers and cognitive profiles although years of education was controlled for. Prospective longitudinal studies will guide more precise insight into these points.

In conclusion, this study demonstrated a hypothetical model of dynamic biomarkers of the pathological cascade of AD by providing both the Aβ burden and neurodegeneration biomarkers in a single PET scan. Aβ depositions reached a plateau first, followed by downstream cerebral perfusion reduction. In addition, both the averaged eFBB and SRTM-based R1 can provide robust indices of cerebral perfusion.

## CRediT authorship contribution statement

**Hai-Jeon Yoon:** Conceptualization, Methodology, Data curation, Formal analysis, Writing - original draft. **Bom Sahn Kim:** Conceptualization, Methodology, Writing - review & editing, Writing - original draft, Supervision. **Jee Hyang Jeong:** Conceptualization, Methodology, Writing - review & editing, Writing - original draft, Supervision. **Geon Ha Kim:** Methodology, Data curation, Validation. **Hee Kyung Park:** Methodology, Data curation, Validation. **Min Young Chun:** Methodology, Data curation, Validation. **Seunggyun Ha:** Methodology, Formal analysis, Writing - review & editing, Writing - original draft.

## Declaration of Competing Interest

The authors declare that they have no known competing financial interests or personal relationships that could have appeared to influence the work reported in this paper.
