# Supplementary material for: Dual-phase 18F-florbetaben PET provides cerebral perfusion proxy along with beta-amyloid burden in Alzheimer’s disease
Source: Neuroimage Clin. 2021 Jul 24;31:102773. doi: 10.1016/j.nicl.2021.102773 (PMC8346681; doi:10.1016/j.nicl.2021.102773)

Supplementary Figure 1. Data of the eFBB SUVR in target cortices through the continuum of AD


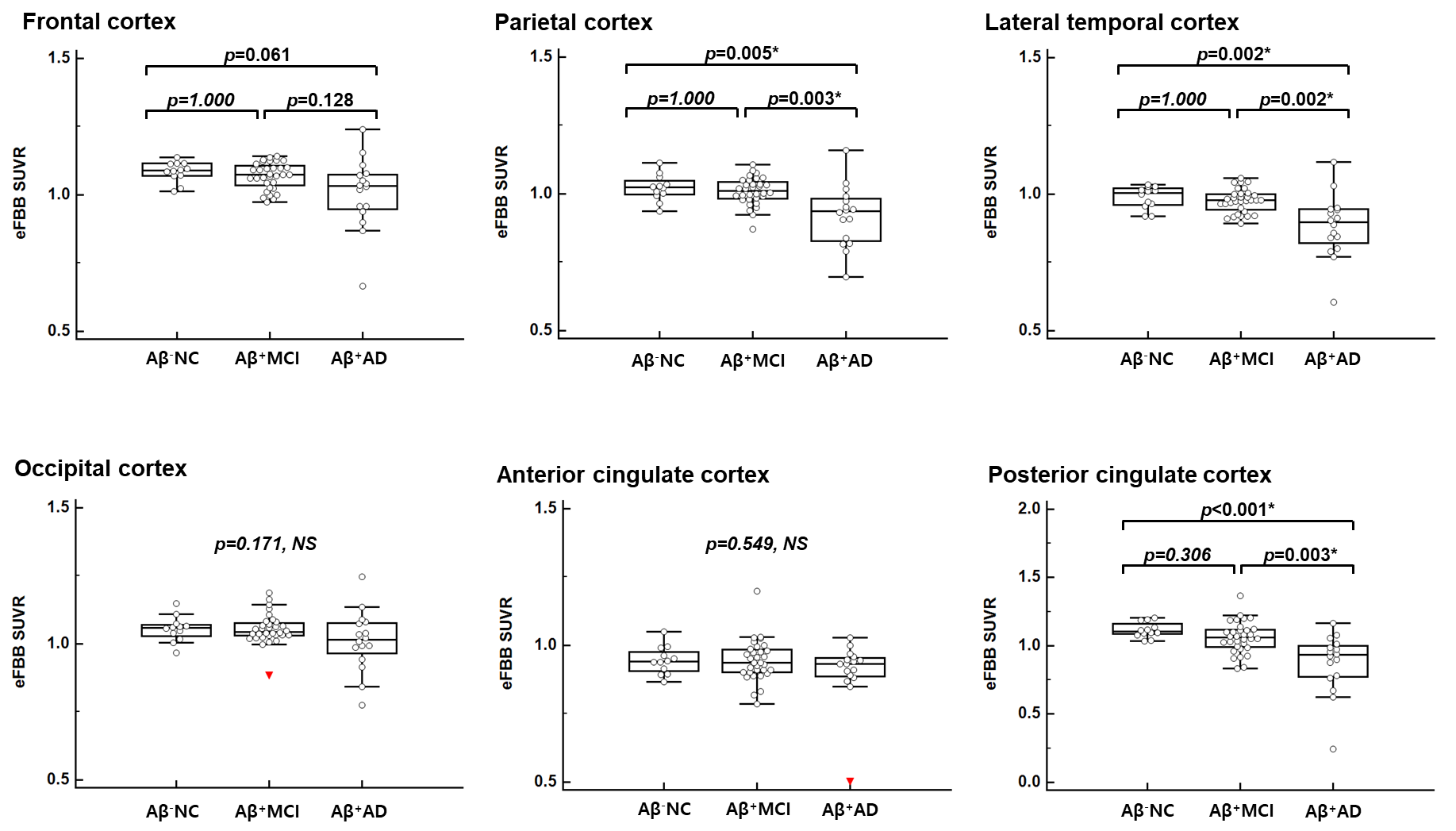


Supplementary Figure 2. Statistical parametric maps of the hypoperfusion patterns obtained from eFBB (0-10 min) images in the Aβ^+^ AD group compared with the Aβ^-^NC group (FDR-corrected *p* < 0.05, *t* > 2.29).


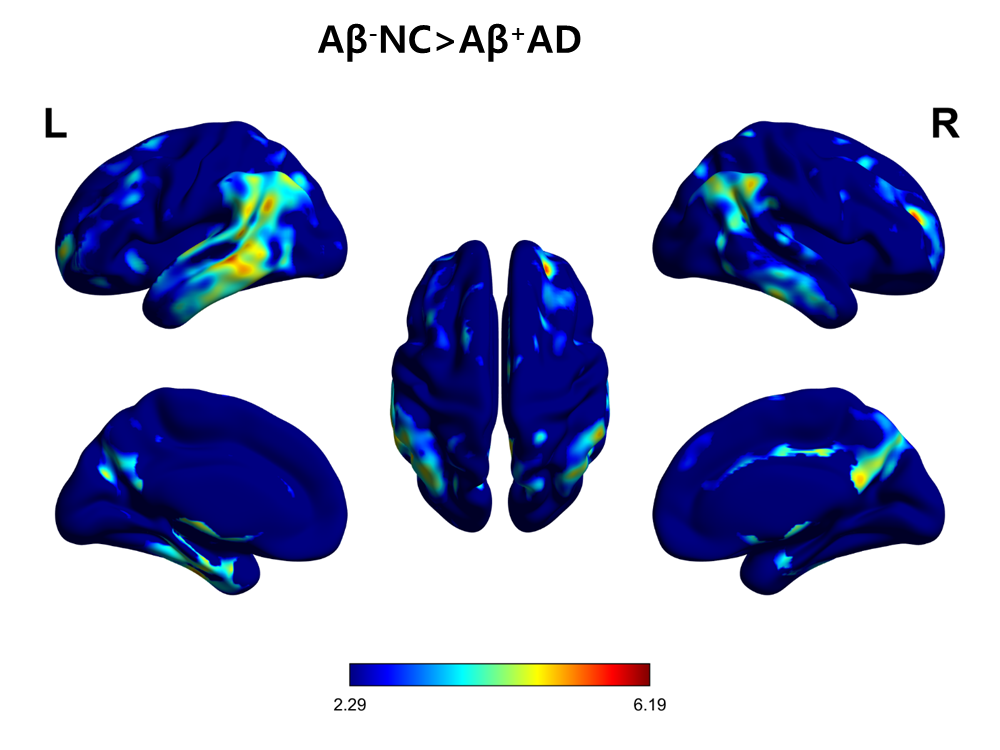


Supplementary Figure 3. Data of the R1 in target cortices through the continuum of AD


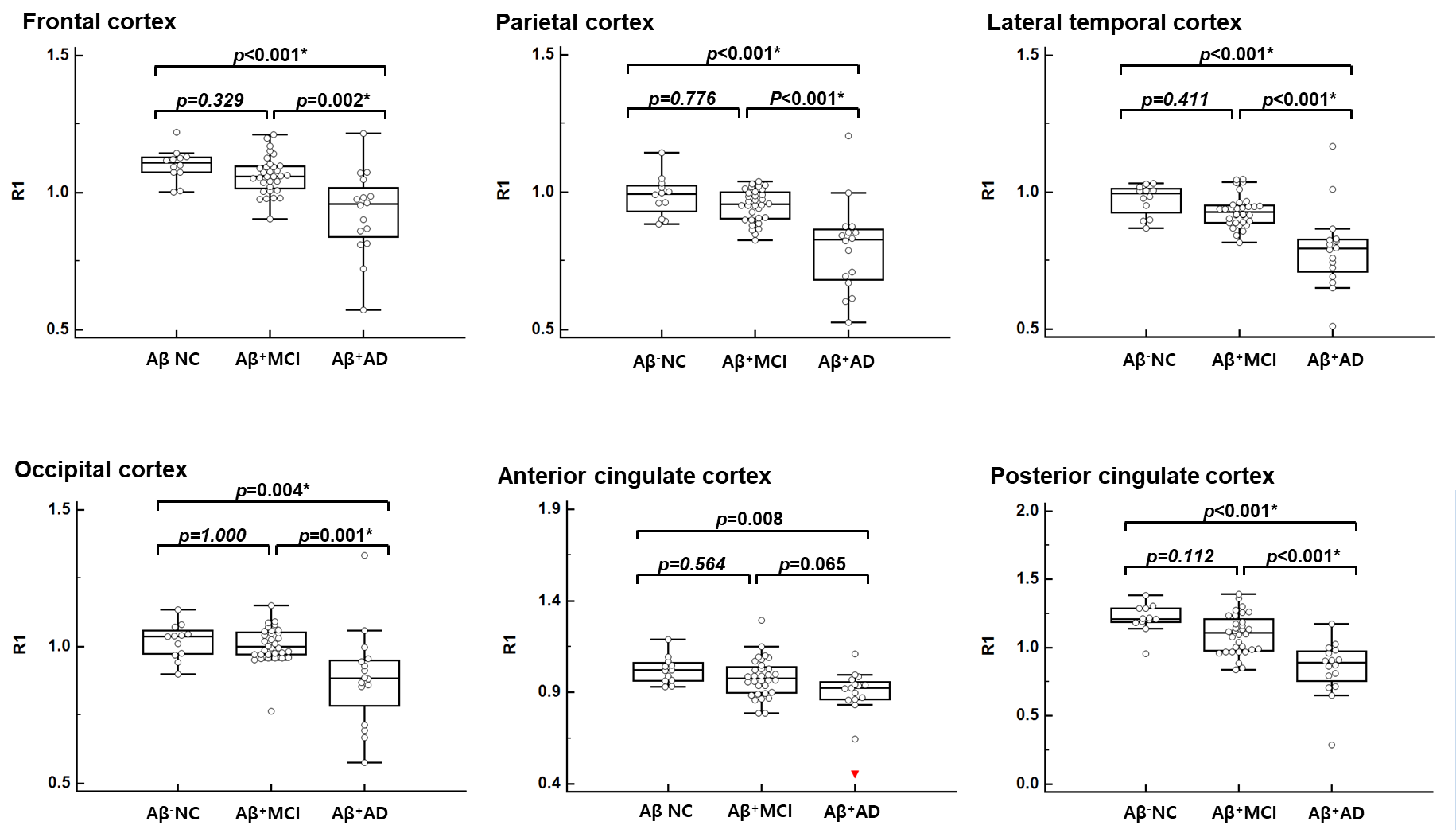


Supplementary Figure 4. Statistical parametric maps of the hypoperfusion patterns obtained from R1 (0-10 min) images in the Aβ^+^ AD group compared with the Aβ^-^NC group (FDR-corrected *p* < 0.05, *t* > 2.33).


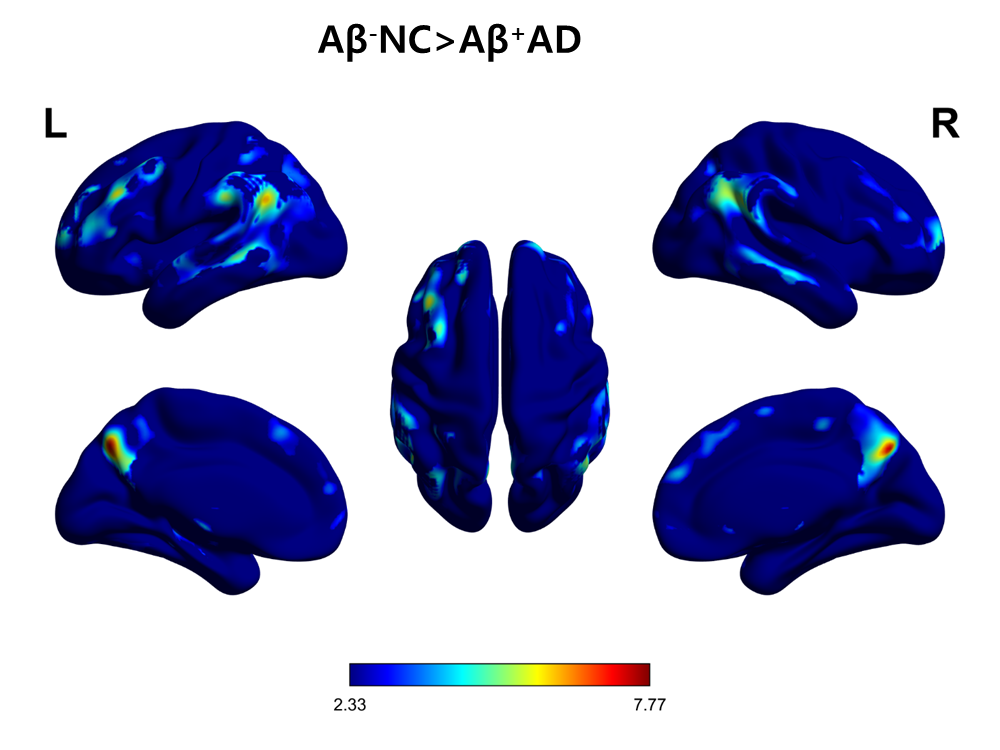


Supplementary Figure 5. Data of the dFBB SUVR in target cortices through the continuum of AD


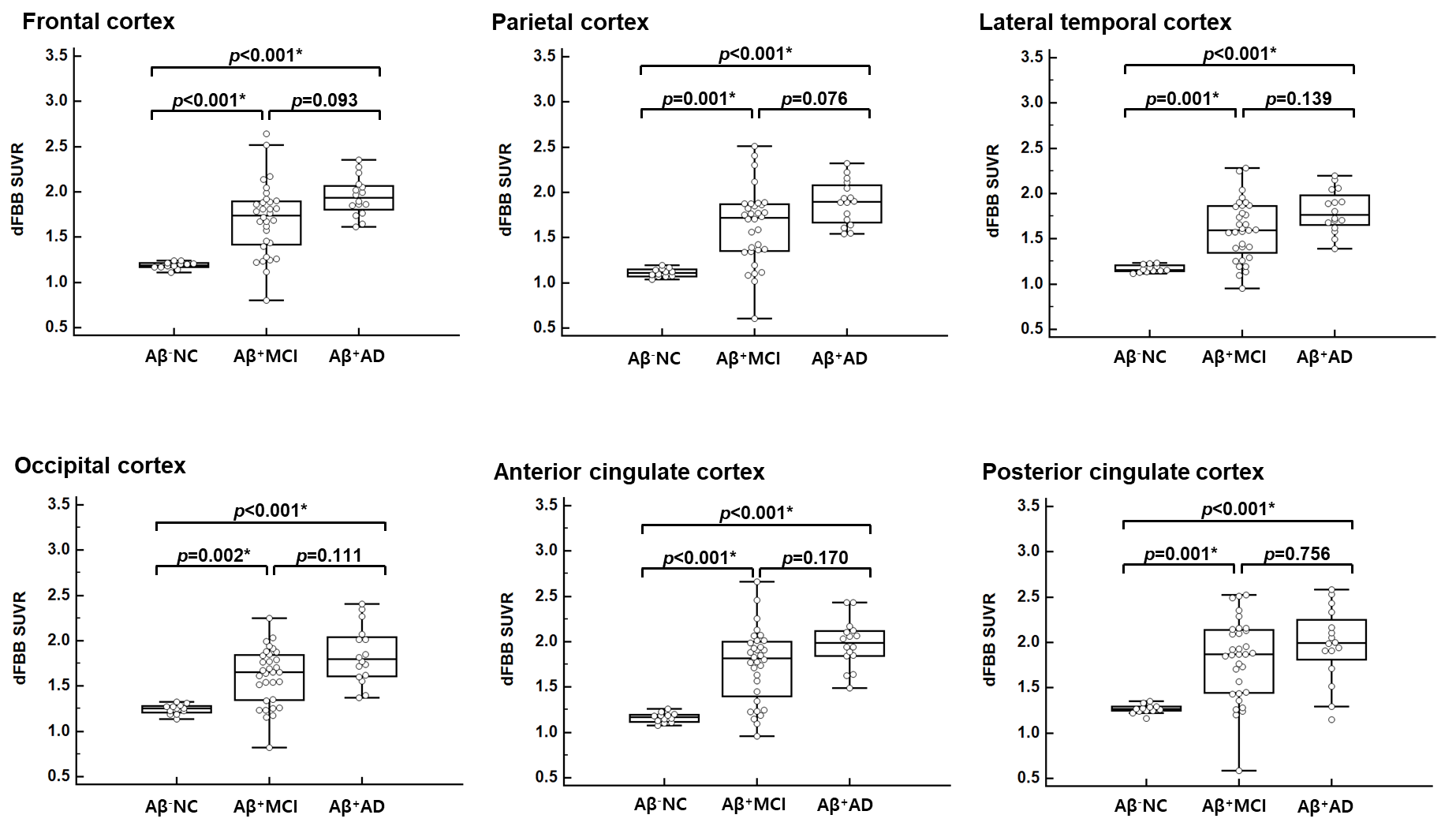


Supplementary Figure 6. VOI-based comparison of R1 between APOE4 noncarriers and carriers in Aβ^+^MCI subgroup


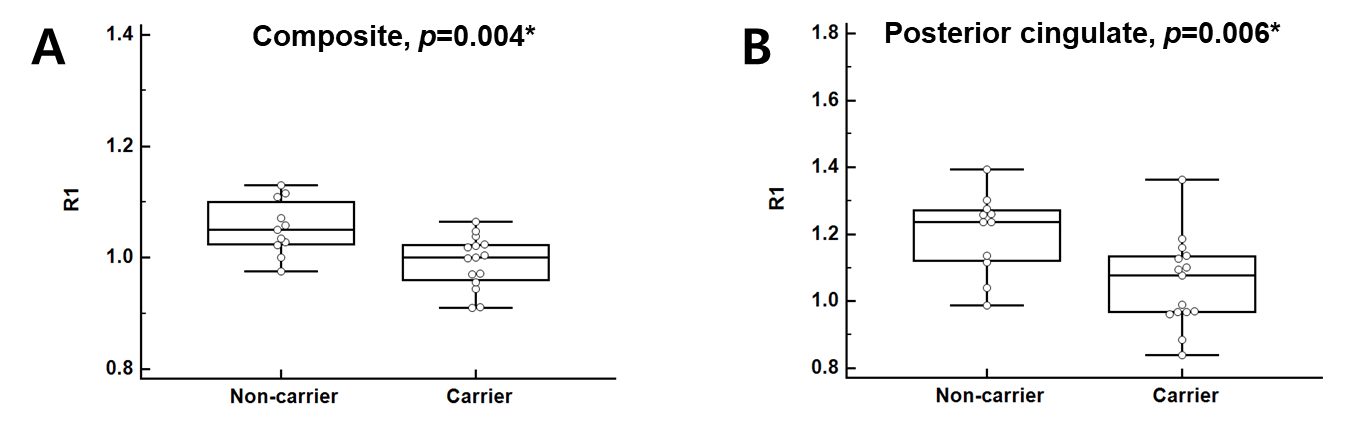


Supplementary Figure 7. VOI-based comparison of dFBB SUVR between APOE4 noncarriers and carriers in Aβ+MCI subgroup


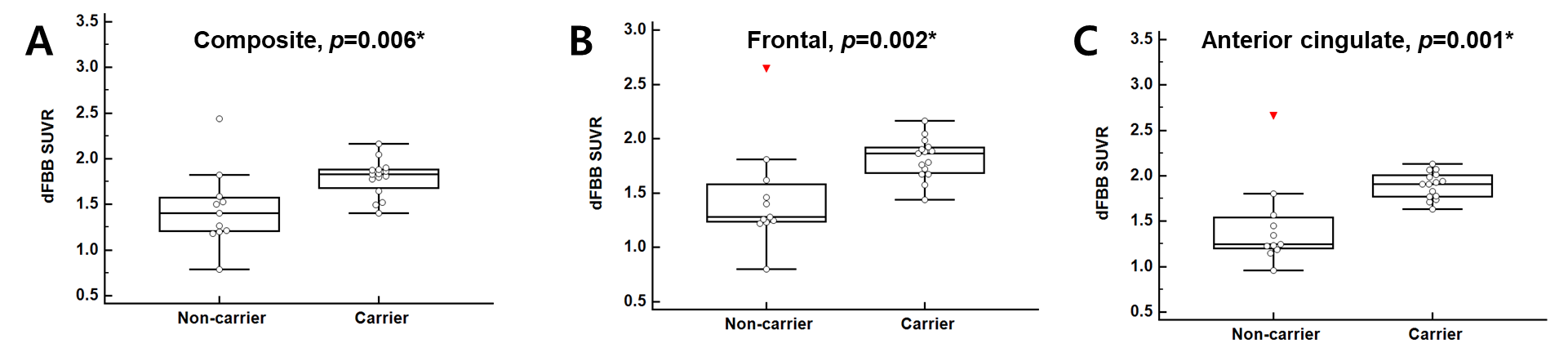

Supplement: Supplementary data 1 [file mmc1.doc]
